# Lectures on the Diagnosis and Treatment of Diseases of the Lungs

**Published:** 1841-02-20

**Authors:** W. W. Gerhard


### MEDICAL EXAMINER.

DEVOTED TO MEDICINE, SURGERY, AND THE COLLATERAL SCIENCES.

No. 8.] PHILADELPHIA, SATURDAY, FEBRUARY 20, 1841. [Vol. IV.

### LECTURES ON THE DIAGNOSIS AND TREATMENT OF DISEASES OF THE LUNGS.

BY W. W. GERHARD, M. D.

### LECTURE XIII.—(*Continued.*)

#### PULMONARY PHTHISIS.

The symptoms of the diseases of other organs than those immediately connected with the lungs, are very numerous in the different periods of pulmonary consumption. Indeed, every disease which produces so deep an impression on the whole economy must of necessity give rise to many functional disorders in the different stages of its progress; and, on the other hand, those local affections will often determine the development of phthisis by the operation of the general laws which we have already laid down as to the connection of tuberculous disease with the enfeebled condition of the body which is readily brought about by the action of a local affection.

When these local symptoms precede phthisis, they are not in most cases dependent upon the development of tubercle; when they occur during the course of the disease, they are more frequently the direct symptoms of the growth and progress of this morbid body, but in the majority of instances this is not the case. The proper way of stating the subject is this:—1. In some cases of tuberculous disease the morbid product is developed in different organs of the body to a sufficient degree to cause its proper symptoms, while the proportion of the tuberculous matter in the lungs is still so much greater than in other viscera, that the specific designation, pulmonary consumption, is retained; in most of these cases the tubercles in the viscera in general, are developed at a later period than those of the lungs; in a few, the former precede the latter. 2. The accompanying disorders and symptoms of other organs than the lungs, which have no immediate connection with the growth of tubercles, are extremely numerous, and occur either previously to phthisis, or in its various stages.

The symptoms of tuberculous disorder of the organs cannot readily be distinguished from those of ordinary chronic inflammation; indeed, the two affections are often united, and occur together. This is particularly the case in the serous membranes; that is, the pia mater, pleuræ, and peritoneum. The inflammation is in these cases of a slow sub-acute variety, and we recognise the tuberculous complication chiefly from its persistence and slow progress. In the intestines the tuberculous disease of the follicles is essentially intermittent at first, and the symptoms vary incessantly, diarrhœa often occurring for several days, and then being followed by constipation; after a certain time the diarrhœa may entirely cease, and the follicles, which are the seat of the tuberculous deposition, will cicatrize. There are no other cases of tuberculous deposit in mucous membranes, in which we can recognise its existence. In the serous membranes, it is essentially connected with inflammation; hence the symptoms are inflammatory, but of the sub-acute kind. All the varieties are closely allied together, and constitute the tuberculous disease of serous membranes which may occur before any tubercles are formed in the lungs, but in the majority of cases they occur in adults during the progress of pulmonary phthisis. In other cases of tuberculous deposit than those just mentioned, the lesion is attended with symptoms of functional disorder of the organs attacked, in proportion as it produces a positive destruction of the tissue, or as it is accompanied with inflammatory action. We see, therefore, that the tuberculous deposit gives rise to few symptoms, except it is so situated as to disturb the function of an organ.

The other lesions, and the attending symptoms which occur in phthisis, or before tubercles are actually formed, are extremely numerous, and very various in character. They are sometimes prominent enough to attract attention almost exclusively to them, and these obscure the characters of the most important affection. Of this nature is dyspepsia, which is a very frequent, though extremely irregular symptom. In some cases it occurs very early in the disorder, and may appear before there is either positive or probable evidence of tuberculous formation; there are cases of dyspeptic phthisis, in which the disorder of the stomach appears often to be quite independent of either general or local tuberculous disease; but in other cases the gastric disturbance is evident before the local disorder, and is clearly connected with the loss of appetite; it may give rise to phthisis in one of two ways,—either by the febrile excitement which it produces when the disease assumes an acute form, or by the alteration of the fluids which produces a peculiar action of the mucous membrane, and causes a slow softening and destruction of it. The complication of dyspepsia and phthisis constitutes one of the worst forms of consumption; as long as the digestive functions are unimpaired, the disease is slow in its progress, and attended with little suffering to the patient; but if the nutrition fails, it becomes much more acute.

The intestinal canal is subject to many derangements; the natural effort of the disorder, like most febrile affections, is to produce constipation; but diarrhœa may occur not only from the formation of tubercles, which has been already mentioned, but from the usual causes of inflammation. In most cases, the symptoms do not differ from those of the same diseases when they occur in a less complicated form; but those of the pulmonary affection are singularly modified, the cough frequently subsides, and the disease is apparently much better. The inflammation of the bowels then acts like any other revulsive action.

Fistula in ano is another affection closely connected with the alimentary canal. Dr. Louis came to the conclusion that this was a rare complication in phthisis, but his conclusions are based upon peculiar data: on examining all the phthisical patients who entered the wards of an hospital, he found that fistula in ano was extremely rare. If he had examined, on the other hand, all cases of fistula in ano admitted into a surgical ward, he would have found that a large proportion of them end in phthisis, either during the continuance of the fistula, or after it has been healed by a surgical operation.

The affections of the liver are frequent in phthisis, especially in women, particularly the young. The most frequent of them is the fatty degeneration of the liver, which is rare, except in phthisis of women and in drunkards. Why these two conditions should both give rise to the same, or nearly the same alteration, is extremely difficult to explain. The functions of the liver are but moderately impaired, notwithstanding a large portion of its tissue should be converted into fat. There is another disease of the liver which occasionally occurs in phthisis, or rather just before the tubercles are developed, which is more important than the fatty state. That is, cyrrhosis: this disorder is most frequent when phthisis occurs in countries where intermittents are endemic, and therefore it is often difficult to distinguish the time when tubercles are formed. The only mode is to attend carefully to the local indications of disease of the lungs, especially the physical signs.

I am compelled to group together the secondary lesions of phthisis, and their symptoms, otherwise this subject would be extended to too great a length. Condensed as this view is, however, some of these secondary altera tions must be omitted, for the very sufficient reason, that a disease of great duration, pervading the whole economy, and causing much febrile excitement, necessarily gives rise to nervous and irregular secondary lesions. Hence we often find that the phthisical patient complains of severe pains in the bones, or muscles, which appear to have no necessary connection with the disease, but belong to the class of unexplained sympathies.

*Diagnosis.*—The diagnosis of phthisis is not attended with any difficulty in advanced, or even in early cases, provided they are regular, and the symptoms follow their usual order. But in cases in which the local signs are not well developed, or the symptoms connected with other organs predominate over those of the lungs, the subject is much more difficult; and we are then obliged to resort to two modes of diagnosis. One is to group together carefully the symptoms we observe, and then to compare with these groups those of other diseases which might possibly give rise to similar symptoms. Thus, any two or three of those symptoms which I have just described as belonging to the lungs, with the addition of emaciation and the febrile movement so frequent in commencing tubercles, would render it probable that the case was one of commencing phthisis. It is true that a complete diagnosis cannot always be made until the disease has advanced far enough to betray some of its essential physical characters, but this is not the case in the majority of patients.

There are certain other signs which are of great value in the diagnosis of early phthisis. These are either individual symptoms, or peculiar modes of formation of collective signs, which would singly be of little value. The most important of them is, perhaps, hæmoptysis. This symptom receives different degrees of attention; some writers consider it almost pathognomonic of phthisis, while others attach comparatively little importance to it. There is, however, little difficulty in reconciling these conflicting opinions; and if we examine the facts relative to it under several points of view, but little real difference of opinion remains. Hæmoptysis occurs in three different relations to phthisis: 1st, before tubercles are developed; 2d, when they are still crude, and perhaps few in number; 3d, when cavities are formed. In the first two cases the blood is evidently secreted from the mucous membrane of the smaller tubes, and probably from the vesicular structure; in the third it comes in most cases from vessels which pass through the bands running across cavities; these may finally give way to ulceration before their caliber is completely obliterated, and a large hæmorrhage may suddenly occur.

Hæmoptysis is of little value as a diagnostic character, unless abundant,—that is, exceeding a wine-glassful in twenty-four hours; a discharge of blood from the lungs in less quantities may, to a certain extent, indicate a tendency to tuberculous disease of these organs, but is not in itself of much importance. If the hæmorrhage be more abundant, and occurs without any obvious cause, it must always be regarded as a sign of commencing phthisis, or of a peculiar condition of the lung itself, or of its capillaries, which often ends in tuberculous formation. The evidence in favour of this conclusion is extremely strong, and is not refuted by the fact that a number of patients affected with hæmorrhage recover; for the first stages of phthisis are by no means incurable; and the varieties in which hæmoptysis occurs are amongst the most favourable. These cases of exemption from phthisis after abundant hæmoptysis are not extremely numerous, as any one may ascertain for himself by simply interrogating individuals who have arrived at the middle periods of life, and enjoy good health: of these a very small proportion have ever had hæmoptysis; and this is true not only with reference to healthy individuals, but as compared with the whole number of phthisical patients; amongst the latter the proportion of cases of hæmorrhage is very large.

The occurrence of tuberculous, or even the long continuance or frequent repetition of simple pleurisy, is another indication of phthisis which will strengthen the more direct symptoms of the disorder. But we must not imagine that any single symptom is ever sufficient for the diagnosis of a disorder, which, at its commencement, is necessarily complex. Nothing but the grouping together of a number of signs, together with indirect evidence afforded by exclusion, will afford the basis of a positive diagnosis.

